# Skeletal muscle index is associated with long term outcomes after lobectomy for non-small cell lung cancer

**DOI:** 10.1186/s12885-023-11210-9

**Published:** 2023-08-19

**Authors:** Yeshwanth Vedire, Lindsay Nitsche, Madeline Tiadjeri, Victor McCutcheon, Jack Hall, Joseph Barbi, Sai Yendamuri, Andrew D. Ray

**Affiliations:** 1 Department of Thoracic Surgery, Roswell Park Comprehensive Cancer Center, Elm and Carlton Streets, Buffalo, NY 14263 USA; 2 Department of Cancer Prevention and Control, Roswell Park Comprehensive Cancer Center, Elm and Carlton Streets, Buffalo, NY 14263 USA; 3 Department of Physical Therapy and Rehabilitation, Roswell Park Comprehensive Cancer Center, Elm and Carlton Streets, Buffalo, NY 14263 USA; 4 Department of Immunology, Roswell Park Comprehensive Cancer Center, Elm and Carlton Streets, Buffalo, Ny 14263 USA; 5 Department of Rehabilitation, Roswell Park Comprehensive Cancer Center, Elm and Carlton Streets, Buffalo, NY 14263 USA

**Keywords:** Non-small cell, Lung cancer, Skeletal muscle, Sarcopenia, Computer tomography

## Abstract

**Background:**

Skeletal muscle indices have been associated with improved peri-operative outcomes after surgical resection of non-small-cell lung cancer (NSCLC). However, it is unclear if these indices can predict long term cancer specific outcomes.

**Methods:**

NSCLC patients undergoing lobectomy at our institute between 2009–2015 were included in this analysis (*N* = 492). Preoperative CT scans were used to quantify skeletal muscle index (SMI) at L4 using sliceOmatic software. Cox proportional modelling was performed for overall (OS) and recurrence free survival (RFS).

**Results:**

For all patients, median SMI was 45.7 cm^2^/m^2^ (IQR, 40–53.8). SMI was negatively associated with age (*R* = -0.2; *p* < 0.05) and positively associated with BMI (*R* = 0.46; *P* < 0.05). No association with either OS or RFS was seen with univariate cox modelling. However, multivariable modelling for SMI with patient age, gender, race, smoking status, DLCO and FEV_1_ (% predicted), American Society of Anesthesiology (ASA) score, tumor histology and stage, and postoperative neoadjuvant therapy showed improved OS (HR = 0.97; *P* = 0.0005) and RFS (HR = 0.97; *P* = 0.01) with SMI. Using sex specific median SMI as cutoff, a lower SMI was associated with poor OS (HR = 1.65, *P* = 0.001) and RFS (HR = 1.47, *P* = 0.03).

**Conclusions:**

SMI is associated with improved outcomes after resection of NSCLC. Further studies are needed to understand the biological basis of this observation. This study provides additional rationale for designing and implementation of rehabilitation trials after surgical resection, to gain durable oncologic benefit.

**Supplementary Information:**

The online version contains supplementary material available at 10.1186/s12885-023-11210-9.

## Background

The long-term survival for Non-Small Cell Lung Cancer (NSCLC) has increased in recent years due to the implementation of more rigorous screening protocols [1]. These protocols that call for a low dose CT scan for patients 50–80 with a 20 pack-year smoking history that currently smoke or quit during that past 15 years [1]. This has facilitated the identification of NSCLC in earlier stages [1]. At these earlier stages the treatment is surgical and advancements in this realm, such as video-assisted (VATS), and robotically-assisted (RATS) thoracoscopic surgery have further contributed to improved outcomes [2, 3]. Concomitantly surgeons have been working to identify patient specific metrics other than DLCO, BMI and VO_2_ peak that can be utilized to predict prognosis and survival. One proposed metric is skeletal muscle index (SMI) obtained by the quantification of skeletal muscle on a CT scan standardized to patient height [4, 5]. Due to its ability to better identify sarcopenia and sarcopenic obesity (presence of low skeletal muscle mass and high BMI concurrently), this metric has more utility than BMI to quantify patient body habitus [5].

Cancer related skeletal muscle loss is multifactorial but is commonly due to age related muscle loss, i.e. sarcopenia, and/or cancer induced hormonal or inflammatory cytokine induced loss, i.e. cachexia [6]. Insufficient physical activity in cancer patients is another common cause of decrease in muscle mass, although this association can be best described as bidirectional. Studies have shown that insufficient exercise in cancer patients leads to a decreased muscle mass, and muscle atrophy which in turn are associated with functional impairment and poor exercise capacity [7, 8]. In a meta-analysis performed by Takenaka et.al which included 2500 cancer patients not only showed significantly poor survival outcomes after immune-checkpoint inhibitor therapy, but also worse objective response and disease control rates [9]. In combination with albumin-globulin score, SMI has been used to predict long term outcomes of patients undergoing resection of intra-hepatic cholangiocarcinoma [10]. Additionally, loss of skeletal muscle mass sometimes may not be reversible by typical nutritional rehabilitation leading to functional impairment [11], frailty [12], reduced chemotherapy tolerance [13], and physical function [14] which in turn can lead to poor peri-operative outcomes.

Since low skeletal muscle mass is prevalent in as many as three quarters of NSCLC patients [15], risk stratification strategies based on SMI have been proposed for patients undergoing thoracoscopic lobectomy [16]. This is consistent with data showing an association between increased thoracic skeletal muscle index (TSMI) and improved peri-operative outcomes in patients undergoing surgery for non-small cell lung cancer (NSCLC) [17]. Increased SMI was correlated with decreased hospital length of stay, fewer complications and decreased mortality in patients undergoing pneumonectomy [17–19]. Despite an association between sarcopenia and OS in all stages of lung cancer, there are conflicting reports suggesting sarcopenia has no effect on relapse free or disease-free survival [19, 20]. The goal of this investigation is to explore the relationship between sarcopenia with OS, and RFS in early-stage lung cancer patients using preoperative CT scans.

The data represents a retrospective analysis of a prospective institutional database.

## Methods

### Ethical statement

This retrospective study was approved by the institutional review board of Roswell Park Comprehensive Cancer Center (RPCCC), Buffalo, New York, USA.

### Clinical data

All patients with NSCLC undergoing lobectomy (Video Assisted Thoracoscopic lobectomy [90%], and open lobectomy [10%]) from 2009–2015 at our institute were included. To avoid confounding by response to chemo or radiotherapy, patients administered neoadjuvant therapy were excluded. A CONSORT diagram is provided as Fig. S1 in Additional file 1 to explain the inclusion and exclusion criteria of the study. Clinical data was extracted from the institutional thoracic surgery database, cancer registry and the electronic health record. Specifically, age, gender, race (White, Black, or other), smoking history (current, former, or never categories), American Society of Anesthesiology (ASA) score, percent-predicted values of diffusion capacity of lung for carbon monoxide (DLCO) and forced expiratory volume in 1 s (FEV_1_) for patients, NSCLC tumor histology (adenocarcinoma, squamous cell carcinoma, or other), pathological stage (as per the 7^th^ edition of the staging manual of the American Joint Committee on Cancer) and survival data (overall and recurrence free survival) was extracted from the cancer registry. The American Society of Anesthesiology (ASA) score was used as a measure of comorbidity and was collapsed into high (score of III/IV) and low (I/II) categories for analysis. Data on subjects receiving adjuvant chemo- and radiotherapy postoperatively was also collected.

### Imaging analysis

Others have shown that imaging software allows for the measurements of single slice CT skeletal muscle (0–150 HU), visceral (-150 – -50 HU), subcutaneous (-190 – -30 HU), and intramuscular adipose tissue (-190 – -30 HU) through the use of tissue-specific Hounsfield Units (HU) ranges as valid and accurate methods to determine whole body composition [21]. Imaging software, sliceOmatic (version 5.0, TomoVision Software, Magog, Canada) was used to quantify the cross-sectional area of muscle (a measure of skeletal muscle mass) at the L4 level. The L4 vertebral level was chosen as it is closer to the center of the psoas muscles (compared to L3), a muscle commonly used to establish sarcopenia with CT scans and also because of the ease of finding it using the umbilicus as an anatomical landmark A skeletal muscle index (SMI) was created by adjusting muscle area for patient height (calculated by dividing the muscle area at L4 by patient height in meters squared) as a measure of body skeletal muscle mass. This adjustment was completed to enable comparisons using the sex specific median SMIs to classify patients into ‘High’ and ‘Low’ categories.

### Statistical analysis

Two-group and three-group comparisons were performed using the Wilcoxon rank sum test and Kruskal-Wallis rank sum test, respectively. Survival analyses was performed to examine associations between overall (OS) and recurrence free (RFS). Univariate and multivariable analyses were performed using the method and Cox proportional Hazards methods, respectively. An alpha error of 0.05 was used to assess statistical significance. As established cutoffs of SMI do not exist, SMI was used as a continuous variable as well as a categorical variable. For multivariable modeling, age, gender, race, smoking status, histology, stage, DLCO, FEV_1_, ASA, post-lobectomy adjuvant chemo and radiotherapy were included in model generation. R and Prism (ver. 9.3.1 for Windows OS, GraphPad Software, San Diego, CA) were used for graphing and to perform analyses.

## Results

After database analysis guided by inclusion and exclusion criteria, a cohort of 492 patients with NSCLC were further evaluated. The median age of the cohort at the time of surgery was 68.5 years (Inter Quartile Range = 61–75). There were 285 (58%) females in our cohort, among them 441 (90%) were Caucasians, and 41 (8.3%) were African American. A higher proportion of the patients were either former (65%) or current (25%) smokers and the median predicted DLCO and FEV1 of the cohort was 76 (63–90) and 80 (65–95), respectively. Adenocarcinoma (66%) was the most common NSCLC histology followed by squamous cell carcinoma (30%). Majority of the patients were diagnosed at AJCC pathological stage I (65%) followed by stage II (27%). The cohort almost had an equal proportion of patients with high (51%) and low (49%) ASA scores. A significant proportion of patients did not receive any adjuvant chemotherapy (73%) or radiation (94%) after surgery. The median BMI was 27 kg/m^2^ (23.9–30.5), 336 (68%) patients had a BMI ≥ 25 kg/m^2^ and were categorized as overweight. The median SMI of the cohort calculated closest to the surgery date was 45.7 cm^2^/m^2^ (40–53.8). The median SMI in males and females was 52.8 cm^2^/m^2^ (46.2–61.5) and 41.9 cm^2^/m^2^ (37.1–46.7), respectively. During follow up 131 (27%) patients developed recurrence, whereas 210 (42.4%) patients died. The median OS and RFS were 65 (37–86) and 56 (24–83) months, respectively. Characteristics of the cohort are summarized in Table 1.

**Table 1:** Characteristics of patients in the study

*Characteristic*	*n (%)*	*SMI*^*1*^	*P value*^*2*^
**Sex**			**< 0.001**
Female	285 (58%)	42 (37–47)	
Male	207 (42%)	53 (46–62)	
**Race**			0.5
Caucasian	441 (90%)	46 (40–54)	
African American	41 (8.3%)	46 (41–57)	
Other	10 (2.0%)	47 (39–49)	
**Smoking**			0.4
Current	124 (25%)	45 (41–55)	
Past	318 (65%)	46 (40–54)	
Never	50 (10%)	45 (39–51)	
**Histology**			0.8
Adeno	324 (66%)	46 (40–53)	
Other	20 (4.1%)	46 (42–50)	
SCC	148 (30%)	46 (39–58)	
**Stage**			0.9
I	321 (65%)	46 (40–54)	
II	133 (27%)	46 (40–56)	
III	38 (7.7%)	46 (40–53)	
**Overweight BMI**			**< 0.001**
Yes	336 (68%)	49 (42–57)	
No	156 (32%)	42 (36–46)	
**ASA score**			0.3
Low (I/II)	241 (49%)	46 (41–55)	
High (III/IV)	251 (51%)	46 (39–53)	
**Chemotherapy**			**0.014**
Yes	132 (27%)	48 (41–58)	
No	360 (73%)	45 (40–53)	
**Radiation**			0.8
Yes	30 (6.1%)	47 (41–54)	
No	462 (94%)	46 (40–54)	
**Recurrence**			0.5
No	361 (73%)	46 (40–54)	
Yes	131 (27%)	46 (40–53)	
**Death**			0.2
No	282 (57%)	46 (41–55)	
Yes	210 (43%)	45 (39–53)	

SMI was compared across the demographic and clinical variables using Wilcoxon rank sum test and Kruskal Wallis rank sum test for two and three groups respectively

*Adeno* Adenocarcinoma, *ASA* American society of anesthesiology, *BMI* Body mass index, *IQR* Inter Quartile Range, *SCC* Squamous cell carcinoma, *SMI* Skeletal muscle index

^1^Median (IQR)

^2^Wilcoxon rank sum test; Kruskal-Wallis rank sum test

### Association of SMI with various demographic and clinical variables

**Fig. 1:**
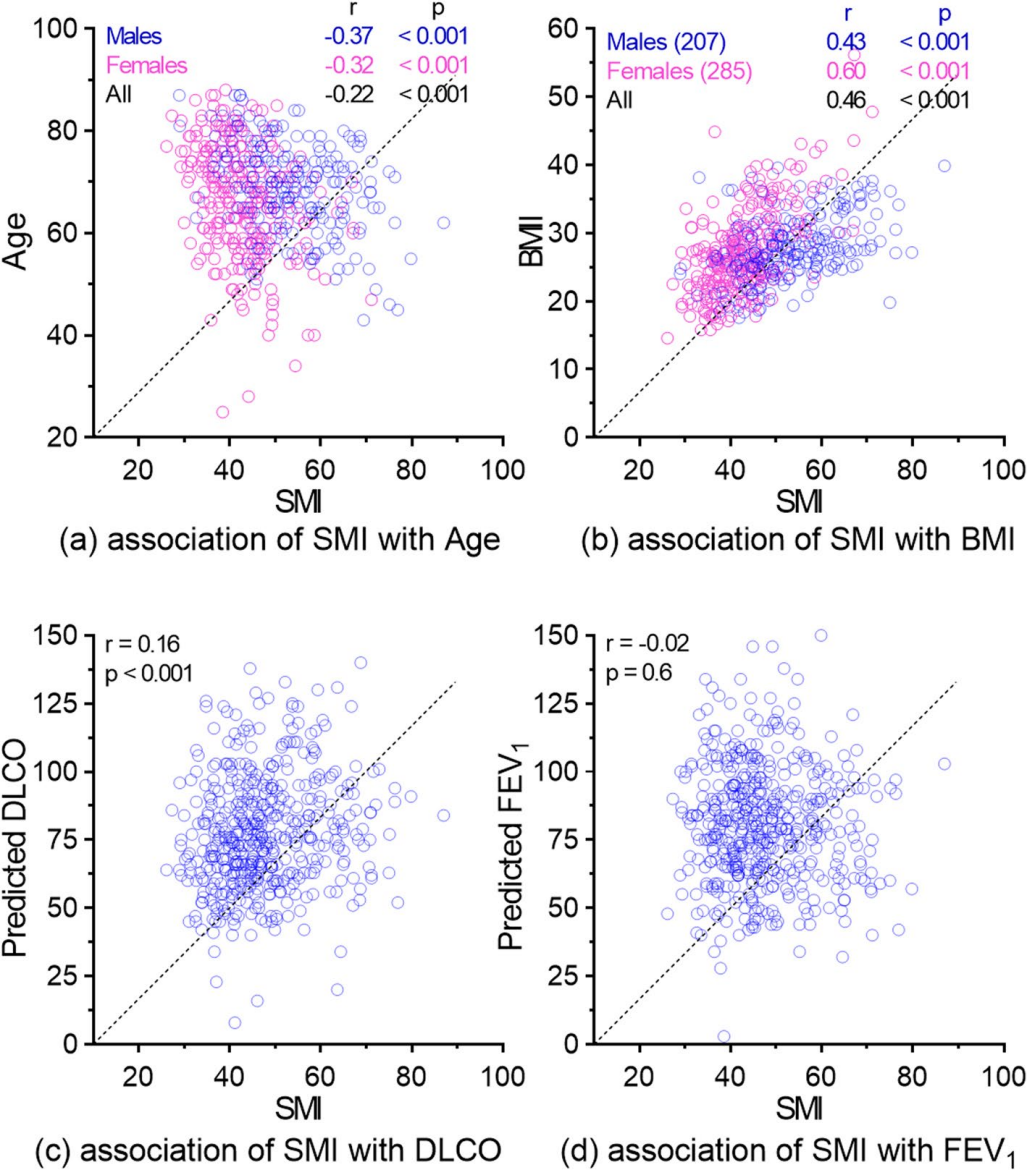
Association of skeletal muscle index (SMI) with age, body mass index (BMI, diffusion capacity of lung for carbon monoxide, and forced expiratory volume at 1 s. SMI was quantified at L4 vertebral level using computerized tomography scans of 492 NSCLC patients. The scatter plots illustrate the correlation between patients’ age, BMI, DLCO, and FEV_1_ and SMI. Pearson correlation coefficients (r) are shown

Male patients had a significantly higher SMI compared to female patients (Median [IQR], 53 [46–62] vs. 42 [37–47]; Wilcoxon rank sum test *p* < 0.001). The median SMI of males was 26% higher compared to the female subgroup. This difference in SMI can be explained by a 47% higher L4 skeletal muscle area. The male and female subgroups did not differ significantly for race (*p* = 0.9) but did so for age (*p* = 0.02), smoking status (*p* = 0.03), and BMI (*p* = 0.01). The higher SMI in males remained significant after adjusting for age, smoking status, and BMI (linear regression β coefficient = 53.56; standard error = 6.45). There was no significant difference in SMI with patient race (Kruskal-Wallis rank sum test *p* = 0.5), smoking status (*p* = 0.4), ASA score (*p* = 0.3), NSCLC tumor histology (*p* = 0.8), stage (*p* = 0.9). Patients who received adjuvant chemotherapy (48 [41–58] vs. 45 [40–53]; *p* = 0.01) but not radiation (*p* = 0.8) had a significantly higher SMI. There was no significant correlation between SMI and FEV_1_. However, there was a modest negative correlation for SMI with age (Pearson *R* = -0.22; *P* < 0.001) and a positive correlation with BMI (*R* = 0.47; *P* < 0.001), and DLCO (*R* = 0.18, *P* < 0.001) (Fig. 1). Similarly, patients with an overweight BMI had a significantly higher SMI compared to normal patients (49 [42–57] vs. 42 [36–46]) (Table S1, Additional file 1)). In multiple linear regression modelling for multivariable analysis, age, gender, BMI, and smoking status but not race were found to be independently associated with SMI with β coefficients of -0.24 (Wald *p* < 0.001), -11.35 (male vs. female, *p* < 0.001), 0.81 (*p* < 0.001) and 2.49 (former vs. current, *p* = 0.002) respectively.

### Survival analysis of NSCLC patients with variations in SMI

Univariate time to event analysis using cox proportional modelling showed that OS was associated with age, sex, BMI, percent-predicted values of FEV_1_ and DLCO, ASA score, NSCLC tumor histology, and stage. Whereas, only patient age, tumor stage and receiving adjuvant chemotherapy were associated with RFS. However, SMI as a continuous variable was not associated with either OS or RFS. However, multivariable modelling with all available patient characteristics as covariates, showed that SMI, age, sex, percent-predicted value of DLCO, and tumor stage were independent predictors for OS. The hazard ratio (HR) for OS was 0.97 (95% CI = 0.95–0.98) and 0.73 for every 1 and 10 cm^2^/m^2^ increase in SMI. Similar multivariable modelling for RFS retained only SMI, sex, and tumor stage as independent predictors with HR of 0.97 (95% CI = 0.95–0.99) and 0.76 for every 1 and 10 cm^2^/m^2^ increase in SMI (Table 2).

**Table 2:** Results of univariate associations and multivariable Cox models of overall and recurrence free survival in NSCLC patients

*Variable*	*Univariate HR (95% CI)*		*Multivariable HR (95% CI)*	
	Overall survival	Recurrence free survival	Overall survival	Recurrence free survival
**Age**	**1.04 (1.03–1.06)**	1.01 (0.99–1.03)	**1.03 (1.01–1.05)**	
**Gender**	**0.52 (0.39–0.68)**	0.71 (0.50–1.00)	**0.31 (0.21–0.46)**	**0.5 (0.31–0.81)**
Female vs. Male				
**Race**				
African American vs. Caucasian	0.59 (0.31–1.03)	0.55 (0.23–1.09)		
Other vs. Caucasian	1.27 (0.45–2.77)	0.75 (0.12–2.38)		
**DLCO**	**0.98 (0.98–0.99)**	0.99 (0.98–1.00)	**0.98 (0.97–0.99)**	
**FEV**_**1**_	**0.99 (0.98–0.99)**	0.99 (0.98–1.00)		
**ASA score**	**1.94 (1.47–2.58)**	1.32 (0.93–1.87)	**1.35 (1.00- 1.82)**	
High vs. Low				
**SMI**	0.98 (0.97–1.00)	0.99 (0.97–1.00)	**0.96 (0.95–0.98)**	**0.97 (0.95–0.99)**
**BMI**^**2**^	1.14 (0.85–1.52)	1.21 (0.84–31.0)		
**Tumor stage**				
Stage II vs. Stage I	1.28 (0.94–1.73)	**2.18 (1.51–3.13)**		**1.87 (1.17–2.96)**
Stage III vs. Stage I	**1.72 (1.06–2.65)**	1.74 (0.89–3.10)	**2.2 (1.17–3.98)**	
**Histology**				
Squamous Cell Carcinoma vs. Adenocarcinoma	**1.57 (1.17–2.09)**	0.97 (0.65–1.42)		
Other vs. Adenocarcinoma	1.9 (0.99–3.30)	1.75 (0.78–3.39)		
**Smoking status**				
Current vs. Past	0.81 (0.57–1.11)	0.89 (0.58–1.33)		
Never vs. Past	0.79 (0.48–1.23)	1.11 (0.63–1.85)		
**Adjuvant Chemotherapy**	0.84 (0.61–1.14)	**1.61 (1.12–2.29)**		
Yes vs. No				
**Adjuvant Radiation**	0.96 (0.52–1.62)	1.55 (0.81–2.69		
Yes vs. No				

*Adeno* Adenocarcinoma, *ASA* American society of anesthesiology, *DLCO* Diffusion capacity of lung for carbon monoxide, *FEV*_*1*_ Forced expiratory volume at 1 s, *SCC* Squamous cell carcinoma, *SMI* Skeletal muscle index

^1^Multivariable analysis was performed using all variables but only variables that were statistically significant are shown. All statistically significant (*P* < 0.05) variables are in bold

^2^Multivariable analysis for BMI and SMI were performed separately but shown in the same table for ease

**Fig. 2:**
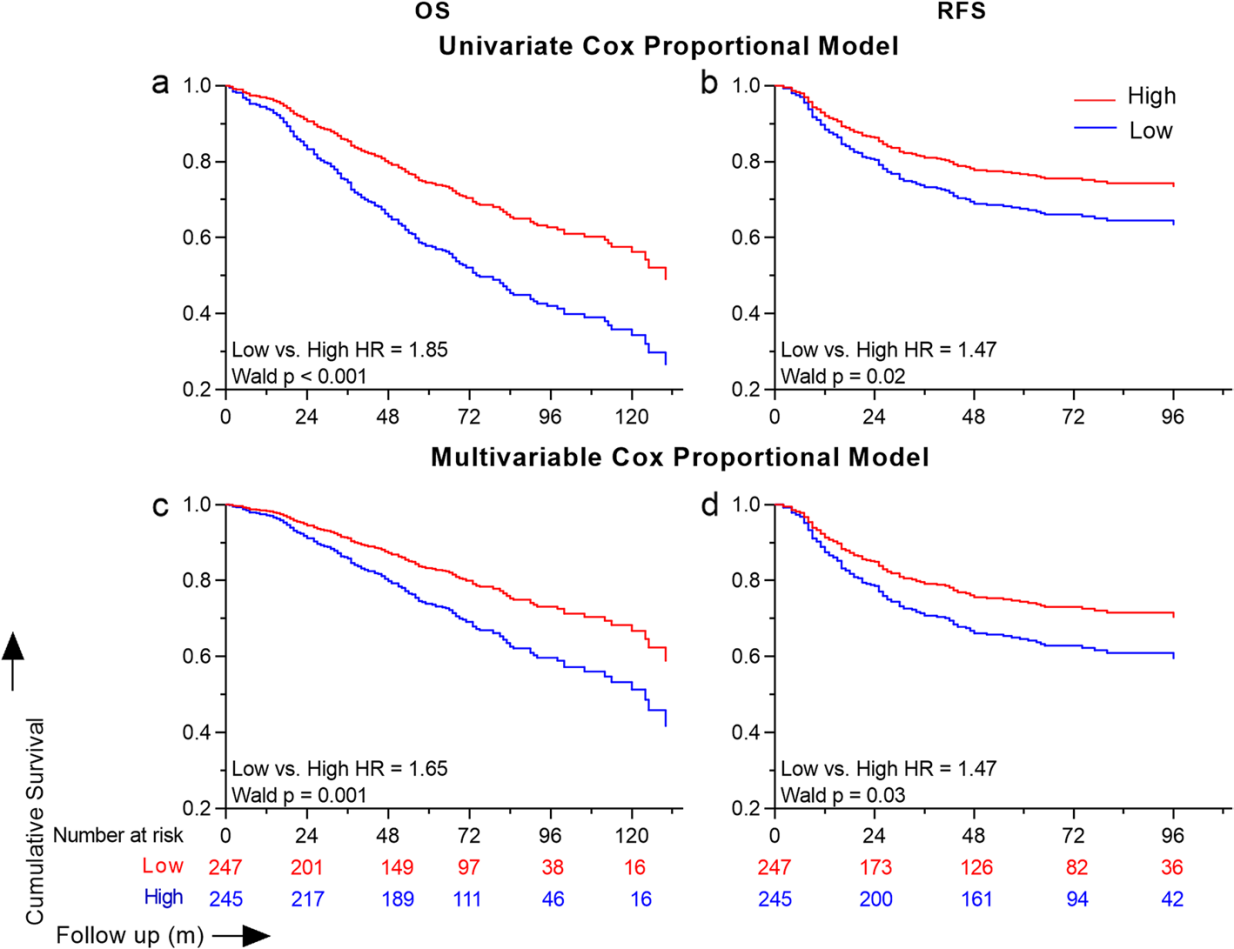
Univariable and multivariable cox proportional models of skeletal mass index (SMI) for overall and recurrence free survival. SMI quantified at L4 vertebral levels was categorized using the sex specific median into ‘High’ (red) and ‘Low’ (blue). The survival curves for univariable and multivariable models for overall and recurrence free survival between the high and low SMI groups are shown. Hazard ratios (HR) and Wald p values are shown

To further understand the association between SMI and post lobectomy outcomes, we categorized SMI based on the median into high and low SMI groups based on their sex specific medians. Univariate cox regression modelling showed an increased HR for both OS (HR = 1.85 [95% CI = 1.41–2.45]) and RFS (HR = 1.47 [95% CI = 1.04–2.09]) with in patients having a low SMI (Fig. 2). In multivariable modelling with all characteristics as covariates, patients with a SMI lower than the median had a significantly poor OS (HR = 1.65 [95% CI = 1.22–2.24]), and RFS (HR = 1.47 [95% CI = 1.02–2.15]).

## Discussion

Our study clearly demonstrates the relationship between skeletal muscle index and long-term oncological outcomes in patients with non-small cell lung cancer. In contrast to the association between SMI and peri-operative outcomes, the association with long term outcomes is not intuitive. Our data demonstrates an association with skeletal muscle index with both overall survival as well as recurrence free survival. Interestingly, this association was not significant on univariate survival analysis, but became significant on multivariable analysis, this may happen due to a few reasons as explained by Lo et al. [22]. Another interesting finding of our study was the lack of relationship between SMI and tumor aggressiveness or stage.

Other investigators have utilized CT imaging to quantify fat and muscle area and make prognostic predictions. This idea was first popularized by Baracos et al., who stressed the secondary utility of oncologic images for body mass composition [23]. Since then, the use of CT images has gained popularity due to their superior image quality, ease of quantification, and a lower patient burden. Recent studies have suggested the use of other metrics such as psoas muscle index (PMI), skeletal muscle gauge (SMG) and skeletal muscle density (SMD) [9]. The use of SMD has been suggested as another indicator of sarcopenia as it is a measure of the quality of skeletal muscle in contrast to SMI which is a measure of quantity. Infiltration of fat into muscle results in low SMD also known as myosteatosis, has also been shown to negatively affect survival [24–27]. While studies have suggested the use of SMI as a static measure [9, 17], to use it as a dynamic variable maybe more apt due to the changes in body composition as a consequence of the underlying cancer and its treatment as suggested by Jang et al. in a meta-analysis. They showed a mean loss of 2.72 cm^2^/m^2^ in SMI of patients during treatment [15].

On a similar note, studies have shown a loss of skeletal muscle to be associated with worse outcomes compared patients who maintained their muscle mass [28–30]. In another study on advanced lung cancer patients, L3 SMI was found to be an independent predictor of malnutrition and a decrease in SMI during chemotherapy was associated with worse 2 year survival [4]. Similar studies in patients undergoing surgery for lung cancer found that a lower preoperative [31] or postoperative [32] skeletal muscle mass was associated with poor outcomes. A study by Kawaguchi et al., using sex specific cut offs for psoas muscle index found that sarcopenic NSCLC patients were more likely to develop postoperative recurrence with a HR of 2.52 (*P* = 0.001) compared to patients without sarcopenia [33]. Our study adds to this burgeoning body of literature suggesting low skeletal muscle mass is an important prognostic factor even in early-stage NSCLC.

Studies have proposed the use of L3 vertebral level SMI as a surrogate marker for sarcopenia and frailty [29, 34, 35]. However, other studies have used total cross-sectional muscle area L1 [36], psoas muscle area specifically at the level of the umbilicus [37], lower pectoralis muscle area [38], and paraspinal muscle index [39] as markers of sarcopenia. In a study Derstine et al., compared the cross sectional muscle area, SMI, and skeletal muscle radiation attenuation between individual vertebral levels from T10 to L5 [5]. The correlation coefficients between all vertebral levels pairs was significant and ranged from 0.64 to 0.95 for SMI, thus defending the use of any of these vertebral levels in its calculation [5]. This study was further corroborated by validating the use of a vertebral level as high as T4 in small cell lung cancer patients [40]. This supports the use of CT scans that optimally capture the thoracic levels for analysis, such as those ordered for NSCLC investigation. SMI has been integrated into algorithms to determine cachexia index in patients and has reliably predicted patients that would have worse PFS and OS showing that it has prognostic value independently and in conjunction with other metrics [41]. Our results support the aforementioned studies suggesting that SMI is associated with improved survival following surgical treatment of NSCLC. However, as compared to previous work, our study has a larger sample size and focuses on SMI as a continuous variable.

An important methodologic issue is the identification of cutoffs for SMI to define sarcopenia. This comes into focus especially given the difference in SMI between males and females. Despite identification of sex-specific cutoffs in the literature [5], we decided to perform our primary analysis using SMI as a continuous variable. The advantage of this approach is the avoidance of bias and assumptions of the interaction between sex and SMI. This is also the reason for selection of the median SMI for analysis. The use of the latter cutoff does come with issues of its own, but it is presented in our results as we feel that it provides useful information notwithstanding its limitations. Larger cohorts for model development and validation will be required for identification of suitable cutoffs for clinical use.

A particular utility of SMI may be in its ability to identify sarcopenic obesity. These patients have a significant depletion of skeletal muscle while concomitantly having an obese-range BMI and are at a higher risk for mortality and morbidity following oncologic treatment. It has been predicted that an average of 9% of cancer patient have sarcopenic obesity with this number being higher in advanced stage cancers [42]. These patients are found to have worse functional status than obese patients without sarcopenia and have worse short- and long-term oncologic outcomes [42, 43]. The molecular mechanisms of how sarcopenic obesity negatively effects the tumor microenvironment are relatively unexplored but maybe due to decreased metabolic rate and increased oxidative stress [43].

The major clinical implication of our study is that physical therapy and nutritional rehabilitation of NSCLC patients undergoing surgery may be an important focus in the peri-operative phase of patient care as observed in a review by Troschel et al. [20]. While physical and nutritional interventions have been shown to improve muscle mass [44], physical function, quality of life while decreasing peri-operative complications [45] in sarcopenic patients, they have not necessarily led to improved long-term outcomes [46, 47]. This maybe because even though interventions halt or reverse muscle loss, improvement in survival outcomes requires a significant reversal of muscle loss which require long-term multimodal interventions [46, 47]. If our observation is borne out by other investigators, this may provide the rationale for the conduct of a randomized controlled trial to assess the impact of such an intervention to achieve durable oncologic benefit. This could potentially take the form of a comprehensive multi-modality approach that optimizes a patient’s nutrition, muscle mass, and physical fitness prior to surgery [47].

## Limitations

Several limitations exist for our study. As it is a retrospective study, the analyses come with all of the attendant biases common to such studies. Other issues include the selection of the L4 level for the measurement of SMI, which may be debated. Also, in the creation of the skeletal muscle index, we did not normalize for other important anthropometric considerations such as gender, race, age, and BMI, all of which may be important for the creation of suitable nomograms. Such considerations can only be addressed by measuring this metric in a considerably larger set of patients and is beyond the scope of this study. Here, we used previously validated imaging methods [21]. Despite these limitations, our findings suggest that further research into the relationship between skeletal muscle mass and cancer recurrence is worthwhile.

### Supplementary Information

**Additional file 1: Table S1.** Characteristics of patients in the study across SMI groups. **Figure S1.** CONSORT diagram form inclusion and exclusion criteria.

## Data Availability

Data generated in the study is available from the corresponding authors upon reasonable request.

## Abbreviations

ASA: American Society of Anesthesiology
BMI: Body mass index
CI: Confidence interval
CT: Computed tomography
DLCO: Diffusing capacity of lung for carbon monoxide
FEV_1_: Forced expiratory volume at 1 s
GEX: Gene expression
HR: Hazard ratio
HU: Hounsfield units
IL: Interleukin
IQR: Interquartile range
NSCLC: Non-small cell lung cancer
OS: Overall survival
OSM: Oncostatin M
PMI: Psoas muscle index
RATS: Robotically-assisted thoracoscopic surgery
RFS: Recurrence free survival
RNA: Ribonucleic acid
SMD: Skeletal muscle density
SMG: Skeletal muscle gauge
SMI: Skeletal muscle index
SPARC: Secreted protein acidic and rich in cysteine
VATS: Video-assisted thoracoscopic surgery
