# Supplementary material for: Skeletal muscle index is associated with long term outcomes after lobectomy for non-small cell lung cancer
Source: BMC Cancer. 2023 Aug 19;23:778. doi: 10.1186/s12885-023-11210-9 (PMC10439565; doi:10.1186/s12885-023-11210-9)
Supplement: Supplementary file 1 — Additional file 1: Table S1. Characteristics of patients in the study across SMI groups. Figure S1. CONSORT diagram form inclusion and exclusion criteria. [file 12885_2023_11210_MOESM1_ESM.docx]

**Supplemental Material**

Skeletal Muscle Index is Associated with Long Term Outcomes After Lobectomy for Non-Small Cell Lung Cancer

Yeshwanth Vedire, Lindsay Nitsche, Madeline Tiadjeri, Victor McCutcheon, John Hall, Robert J. Seager, Sarabjot Pabla, Joseph Barbi, Sai Yendamuri, Andrew Ray.

*Table of Contents*

1. Table S1. *Characteristics of patients in the study across SMI groups*
2. Figure S1. *CONSORT diagram for study inclusion and exclusion criteria.*

**Table S1.** *Characteristics of patients in the study across SMI groups*

| *Characteristic* | *All (n=492)^1^* | *High SMI (n = 246)* | *Low SMI (n = 246)* | *P value^2^* |
| --- | --- | --- | --- | --- |
| Age | 67.47 (10.23) | 65.81 (9.5) | 69.13 (10.6) | **< 0.01** |
| Sex |  |  |  | **< 0.01** |
| Female | 285 (57.9%) | 88 (35.8%) | 197 (80.1%) |  |
| Male | 207 (42.1%) | 159 (64.2%) | 48 (19.9%) |  |
| Race |  |  |  | 0.42 |
| White | 441 (89.1%) | 217 (87.5%) | 224 (90.7%) |  |
| Black | 40 (8.1%) | 24 (9.7%) | 16 (6.5%) |  |
| Other | 14 (2.8%) | 7 (2.8%) | 7 (2.8%) |  |
| DLCO | 77.29 (20.87) | 80.22 (21.1) | 74.29 (20.24) | **< 0.01** |
| ASA score |  |  |  | 0.28 |
| High | 251 (51%) | 122 (49.6%) | 129 (52.4%) |  |
| Low | 241 (49%) | 124 (50.4%) | 117 (47.6%) |  |
| BMI | 27.49 ± 5.63 | 29.6 ± 5.5 | 25.3 ± 4.9 | **< 0.01** |
| Tumor stage |  |  |  |  |
| Stage I | 321 (65.2%) | 159 (64.6%) | 162 (65.9%) | 0.8 |
| Stage II | 133 (27%) | 66 (26.9%) | 67 (27.2%) |  |
| Stage III | 38 (7.8%) | 21 (8.5%) | 17 (6.9%) |  |
| Histology |  |  |  |  |
| Adenocarcinoma | 324 (65.9%) | 162 (65.9%) | 162 (65.9%) | 0.9 |
| Squamous Cell Carcinoma | 148 (30%) | 74 (30%) | 75 (30%) |  |
| Other | 20 (4.1%) | 11 (4.1%) | 9 (4.1%) |  |
| Smoking |  |  |  | 0.8 |
| Former | 318 (64.6%) | 162 (65.9%) | 156 (63.4%) |  |
| Current | 124 (25.2%) | 60 (24.4%) | 64 (26%) |  |
| Never | 50 (10.2%) | 24 (9.8%) | 26 (10.6%) |  |
| *^1^ Mean (SD); n (%)* | | | | |
| *^2^ 2-tailed standard t test; Fischer's Exact test* | | | | |

High and low SMI groups were separated by calculating the median. Standard t test and Fischer’s Exact test were used to compare continuous and categorical clinical and demographic variables between high and low SMI groups. All statistically significant (P < 0.05) variables are in bold.

ASA, American society of anesthesiology; DLCO, diffusion capacity of lung for carbon monoxide; SD, standard deviation; SMI, skeletal muscle index

**Figure S1.** CONSORT Diagram for study inclusion and exclusion criteria.

**Cohort 1**

**Exclusion Criteria**

L4 level CT image not available within 1 year of surgery date (n = 0)

Final cohort size of 492 patients

Cohort of 492 patients

Stage I/II/III NSCLC patients undergoing lobectomy between 2009-15 at our institute
